# Supplementary material for: Polyketide synthase gene clusters in the ericoid mycorrhizal fungus Oidiodendron maius: genome-wide prediction, expression profiling, and functional characterization of knockout mutants
Source: Front Plant Sci. 2026 Sep 16;17:1924430. doi: 10.3389/fpls.2026.1924430 (PMC13623903; doi:10.3389/fpls.2026.1924430)
Supplement: Supplementary file 1 [file SupplementaryFile1.zip › Supplementary Materials/Supplementary Folder 1.zip!/Oidma1_AssemblyScaffolds_Repeatmasked/knownclusterblast/cluster38/jgi.p_Oidma1_133211_mibig_hits.html]

| MIBiG Protein | Description | MIBiG Cluster | MiBiG Product | % ID | % Coverage | BLAST Score | E-value |
| --- | --- | --- | --- | --- | --- | --- | --- |
| AHN91924.1 | EchA | BGC0000340 | Echosides\_biosynthetic\_gene\_cluster | 37 | 93.137254902 | 557 | 3.7e-158 |
| CBF80711.1 | TdiA\_[Source:UniProtKB/TrEMBL;Acc:A7XRY0] | BGC0000442 | Terrequinone\_biosynthetic\_gene\_cluster | 35 | 103.48583878 | 527 | 4.1e-149 |
| ACH90386.1 | atromentin\_synthetase | BGC0001256 | Atromentin\_biosynthetic\_gene\_cluster | 33 | 96.6230936819 | 504 | 2.9e-142 |
| ABM21569.1 | CrpA | BGC0000975 | Cryptophycin\_biosynthetic\_gene\_cluster | 30 | 66.9934640523 | 272 | 1.4e-72 |
| AAN32979.1 | BarE | BGC0000962 | Barbamide\_biosynthetic\_gene\_cluster | 29 | 68.082788671 | 262 | 2.5e-69 |
| AAO62585.1 | peptide\_sythetase\_polyketide\_synthase\_fusion\_protein | BGC0001016 | Microcystin\_biosynthetic\_gene\_cluster | 29 | 69.1721132898 | 261 | 4.2e-69 |
| AHJ31217.1 | Long-chain-fatty-acid--CoA\_ligase | BGC0000430 | Spumigin\_biosynthetic\_gene\_cluster | 33 | 53.48583878 | 255 | 1.8e-67 |
| AAF00957.1 | McyG | BGC0001017 | Microcystin\_biosynthetic\_gene\_cluster | 30 | 63.834422658 | 253 | 8.8e-67 |
| ABM21572.1 | CrpD | BGC0000975 | Cryptophycin\_biosynthetic\_gene\_cluster | 33 | 56.2091503268 | 250 | 9.7e-66 |
| AEU11005.1 | NpnA | BGC0001029 | Nostophycin\_biosynthetic\_gene\_cluster | 31 | 57.1895424837 | 248 | 2.8e-65 |
| CAD29795.1 | peptide\_synthetase | BGC0001015 | Microcystin\_biosynthetic\_gene\_cluster | 27 | 70.2614379085 | 243 | 7e-64 |
| AAY42398.1 | HctF | BGC0001000 | Hectochlorin\_biosynthetic\_gene\_cluster | 31 | 56.3180827887 | 241 | 3.5e-63 |
| AAY42397.1 | HctE | BGC0001000 | Hectochlorin\_biosynthetic\_gene\_cluster | 31 | 56.3180827887 | 237 | 5e-62 |
| ABD14712.1 | CesB | BGC0000320 | Cereulide\_biosynthetic\_gene\_cluster | 29 | 59.1503267974 | 230 | 8e-60 |
| CAM59601.1 | non-ribosomal\_peptide\_synthetase/polyketide\_synthase\_hybrid\_enzyme | BGC0000297 | Aeruginoside\_biosynthetic\_gene\_cluster | 29 | 58.7145969499 | 229 | 1.4e-59 |
| AKA54626.1 | NRPS | BGC0001216 | Splenocin\_biosynthetic\_gene\_cluster | 30 | 54.0305010893 | 221 | 3.7e-57 |
| ACM68682.1 | AerA | BGC0000298 | Aeruginosin\_biosynthetic\_gene\_cluster | 28 | 59.0413943355 | 215 | 3.5e-55 |
| WP\_010639240.1 | gramicidin\_synthetase | BGC0000958 | Antimycin\_biosynthetic\_gene\_cluster | 29 | 54.0305010893 | 213 | 1e-54 |
| AEF33080.1 | putative\_peptide\_synthetase | BGC0001039 | Pyridomycin\_biosynthetic\_gene\_cluster | 30 | 55.7734204793 | 212 | 1.7e-54 |
| AFK57217.1 | DidF | BGC0000985 | Didemnin\_biosynthetic\_gene\_cluster | 31 | 53.48583878 | 209 | 1.5e-53 |
| ABD14711.1 | CesA | BGC0000320 | Cereulide\_biosynthetic\_gene\_cluster | 27 | 58.9324618736 | 204 | 4.7e-52 |
| ABY83140.1 | Azi1 | BGC0000960 | Azinomycin\_B\_biosynthetic\_gene\_cluster | 29 | 56.1002178649 | 183 | 1.1e-45 |
| BAB69698.1 | iturin\_A\_synthetase\_A | BGC0001098 | Iturin\_biosynthetic\_gene\_cluster | 24 | 74.2919389978 | 182 | 2.5e-45 |
| ABS74181.1 | BmyA | BGC0001090 | Bacillomycin\_biosynthetic\_gene\_cluster | 24 | 74.5098039216 | 182 | 2.5e-45 |
| CBZ42145.1 | non-ribosomal\_peptide\_synthetase | BGC0001117 | Himastatin\_biosynthetic\_gene\_cluster | 26 | 60.1307189542 | 170 | 7.5e-42 |
| AID65222.1 | putative\_aspartate\_racemase | BGC0000335 | Cystomanamides\_biosynthetic\_gene\_cluster | 24 | 72.440087146 | 163 | 1.2e-39 |
| AAF08795.1 | MycA | BGC0001103 | Mycosubtilin\_biosynthetic\_gene\_cluster | 24 | 71.568627451 | 163 | 1.2e-39 |
| ALV86868.1 | Tlo22 | BGC0001406 | Telomycin\_biosynthetic\_gene\_cluster | 24 | 71.3507625272 | 139 | 1.9e-32 |
| BAH22765.1 | nonribosomal\_peptide\_synthetase | BGC0001018 | Micropeptin\_biosynthetic\_gene\_cluster | 21 | 85.0762527233 | 129 | 1.9e-29 |
| ACM79812.1 | ZmaQ | BGC0001059 | Zwittermycin\_A\_biosynthetic\_gene\_cluster | 23 | 67.4291938998 | 129 | 2.5e-29 |
| AAZ03554.1 | McnE | BGC0000332 | Cyanopeptolin\_biosynthetic\_gene\_cluster | 21 | 85.5119825708 | 126 | 1.6e-28 |
| CAE02633.1 | surfactin\_synthetase\_C | BGC0000433 | Surfactin\_biosynthetic\_gene\_cluster | 24 | 66.1220043573 | 124 | 8e-28 |
| ADJ63842.1 | non-ribosomal\_peptide\_synthetase\_protein | BGC0000424 | Serobactins\_biosynthetic\_gene\_cluster | 26 | 55.6644880174 | 116 | 1.3e-25 |
| ABW17377.1 | PsoC | BGC0000411 | Putisolvin\_biosynthetic\_gene\_cluster | 24 | 67.3202614379 | 113 | 1.9e-24 |
| AAQ84158.1 | PlmJK | BGC0000123 | Phoslactomycin\_B\_biosynthetic\_gene\_cluster | 28 | 33.7690631808 | 109 | 2.7e-23 |
| AAU39361.1 | lichenysin\_synthase\_LchAC | BGC0000381 | Lichenysin\_biosynthetic\_gene\_cluster | 21 | 66.0130718954 | 108 | 4.6e-23 |
| AGD80622.1 | AMP-dependent\_synthetase\_and\_ligase | BGC0000394 | Naphthyridinomycin\_biosynthetic\_gene\_cluster | 24 | 67.3202614379 | 108 | 6e-23 |
| AAC06348.1 | bacitracin\_synthetase\_3 | BGC0000310 | Bacitracin\_biosynthetic\_gene\_cluster | 21 | 69.6078431373 | 103 | 1.5e-21 |
| AJM89734.1 | PmxB | BGC0001192 | Colistin\_biosynthetic\_gene\_cluster | 21 | 66.339869281 | 99 | 2.8e-20 |
| BAO84862.1 | putative\_long-chain-fatty-acid-CoA\_ligase | BGC0000414 | Quinocarcin\_biosynthetic\_gene\_cluster | 26 | 44.7712418301 | 97 | 8.1e-20 |
| AGY30675.1 | Ann3 | BGC0001298 | 4-Z-annimycin\_biosynthetic\_gene\_cluster | 26 | 54.6840958606 | 97 | 8.1e-20 |
| BAO66547.1 | cyclohexanecarboxylate-CoA\_ligase | BGC0000042 | Cremimycin\_biosynthetic\_gene\_cluster | 30 | 31.3725490196 | 94 | 5.2e-19 |
| AEI98652.1 | CtcI | BGC0000209 | Chlortetracycline\_biosynthetic\_gene\_cluster | 28 | 31.5904139434 | 92 | 2.6e-18 |
| BAH33409.1 | putative\_non-ribosomal\_peptide\_synthetase | BGC0000371 | Heterobactin\_biosynthetic\_gene\_cluster | 23 | 42.7015250545 | 90 | 1.3e-17 |
| WP\_010639241.1 | beta-ketoacyl\_synthase | BGC0000958 | Antimycin\_biosynthetic\_gene\_cluster | 28 | 22.0043572985 | 89 | 2.9e-17 |
| AIL50189.1 | putative\_5-aminolevulinate\_CoA-ligase | BGC0000213 | Colabomycin\_biosynthetic\_gene\_cluster | 27 | 41.2854030501 | 86 | 2.4e-16 |
| BAG16281.1 | putative\_2,3-dihydroxybenzoate-AMP\_ligase | BGC0000632 | Brasilicardin\_A\_biosynthetic\_gene\_cluster | 24 | 33.660130719 | 85 | 3.2e-16 |
| ABC35922.1 | AMP-binding\_domain\_protein | BGC0001102 | Malleilactone\_biosynthetic\_gene\_cluster | 24 | 40.1960784314 | 85 | 4.1e-16 |
| AGL76720.1 | fatty\_acid\_CoA\_ligase | BGC0001137 | Marinacarboline\_biosynthetic\_gene\_cluster | 25 | 36.2745098039 | 84 | 9.2e-16 |
| AHD25943.1 | ChdL | BGC0000208 | Chelocardin\_biosynthetic\_gene\_cluster | 27 | 32.2440087146 | 82 | 2.1e-15 |
| ABP55493.1 | thioester\_reductase\_domain | BGC0001006 | Lymphostin\_biosynthetic\_gene\_cluster | 28 | 39.651416122 | 81 | 6e-15 |
| CCC55917.1 | putative\_acyl\_CoA\_ligase | BGC0000973 | Collismycin\_A\_biosynthetic\_gene\_cluster | 26 | 43.3551198257 | 81 | 7.8e-15 |
| AAL65279.1 | QbsL | BGC0000925 | Quinolobactin\_biosynthetic\_gene\_cluster | 31 | 25.1633986928 | 80 | 1.3e-14 |
| ACO31297.1 | PtmA3 | BGC0001140 | Platensimycin\_/\_platencin\_biosynthetic\_gene\_cluster | 26 | 34.6405228758 | 77 | 8.6e-14 |
| AFH75322.1 | nonribosomal\_peptide\_synthetase | BGC0000425 | Sessilin\_biosynthetic\_gene\_cluster | 23 | 38.1263616558 | 77 | 1.1e-13 |
| CBK62739.1 |  | BGC0001115 | Thiomarinol\_biosynthetic\_gene\_cluster | 28 | 33.0065359477 | 76 | 2.5e-13 |
| CCJ67640.1 | TaaE | BGC0000447 | Tolaasin\_biosynthetic\_gene\_cluster | 23 | 46.7320261438 | 75 | 4.3e-13 |
| CAQ52626.1 | type\_I\_polyketide\_synthase,\_loading\_module\_and\_modules\_1-3 | BGC0001066 | Kendomycin\_biosynthetic\_gene\_cluster | 26 | 42.1568627451 | 75 | 4.3e-13 |
| AEW31015.1 | plipastatin\_synthetase | BGC0000407 | Plipastatin\_biosynthetic\_gene\_cluster | 26 | 22.2222222222 | 74 | 7.3e-13 |
| ADD82992.1 | PtnA3 | BGC0001156 | Platencin\_biosynthetic\_gene\_cluster | 26 | 32.4618736383 | 73 | 1.6e-12 |
| AAP92505.1 | nonribosomal\_peptide\_synthetase | BGC0000458 | Viomycin\_biosynthetic\_gene\_cluster | 23 | 53.1590413943 | 71 | 8.1e-12 |
| ADH04679.1 | non-ribosomal\_peptide\_synthetase | BGC0001344 | Tubulysin\_biosynthetic\_gene\_cluster | 21 | 56.7538126362 | 68 | 5.2e-11 |
| ABL74940.1 | TlmVI | BGC0001048 | Tallysomycin\_biosynthetic\_gene\_cluster | 27 | 22.1132897603 | 67 | 6.8e-11 |
| CAC48361.1 | peptide\_synthetase | BGC0000311 | Balhimycin\_biosynthetic\_gene\_cluster | 23 | 39.5424836601 | 67 | 1.2e-10 |
| AFJ23825.1 | WLIP\_synthetase\_B | BGC0000462\_c2 | WLIP\_biosynthetic\_gene\_cluster | 23 | 63.7254901961 | 66 | 1.5e-10 |
| ABF87031.1 | non-ribosomal\_peptide\_synthetase/polyketide\_synthase | BGC0000393 | Myxoprincomide\_biosynthetic\_gene\_cluster | 27 | 22.1132897603 | 66 | 2.6e-10 |
| CAQ71827.1 | non\_ribosomal\_peptide\_synthase,\_antibiotic\_synthesis;\_contains\_1\_condensation\_domain,\_1\_AMP-acid\_ligases\_II\_domain | BGC0001189 | Taiwachelin\_biosynthetic\_gene\_cluster | 22 | 56.1002178649 | 64 | 7.6e-10 |
| AHZ34233.1 | CifB | BGC0000323 | Cichofactin\_biosynthetic\_gene\_cluster | 22 | 35.4030501089 | 62 | 2.2e-09 |
| CAJ96471.1 | Non-ribosomal\_peptide\_synthetase | BGC0000330 | Cupriachelin\_biosynthetic\_gene\_cluster | 23 | 55.8823529412 | 62 | 3.8e-09 |
| CAP96445.1 |  | BGC0000420 | Roquefortine\_C\_/\_meleagrin\_biosynthetic\_gene\_cluster | 24 | 55.7734204793 | 60 | 1.4e-08 |
| BAH23995.1 | nonribosomal\_peptide\_synthetase | BGC0000356 | Fumitremorgin\_biosynthetic\_gene\_cluster | 24 | 41.6122004357 | 59 | 1.9e-08 |
